# Applicability of Food Monitoring Data for Assessing Relative Exposure Contributions of Pyrethroids in Retrospective Human Biomonitoring Risk Estimations

**DOI:** 10.3390/toxics12010024

**Published:** 2023-12-28

**Authors:** Mercedes de Alba-Gonzalez, Maria Carmen González-Caballero, Jose V. Tarazona

**Affiliations:** National Centre for Environmental Health, Instituto de Salud Carlos III, 28220 Madrid, Spain; mcgonzalez@isciii.es (M.C.G.-C.); jtarazona@isciii.es (J.V.T.)

**Keywords:** pyrethroids, urinary levels, DCCA, CFMP, 3PBA, DBCA, F3PBA, CLF3CA, pesticide risk, HBM4EU

## Abstract

The use of pyrethroids is very broad and shows increasing trends. Human biomonitoring studies represent the best approach for realistic risk estimations, but their interpretation requires a tiered approach. A previous HBM4EU study indicated levels in European children groups just around the threshold for concern, requiring further refinement. The main difficulty is that several pyrethroids with different toxicity potencies generate the same urinary metabolites. As diet is the main pyrethroid source for the general population, EU food monitoring data reported by EFSA have been used to estimate the relative contribution of each pyrethroid. The main contributors were cypermethrin for DCCA and 3-PBA and lambda-cyhalothrin for CFMP. Urinary levels predicted from food concentration according to the EFSA diets were mostly within the range of measured levels, except 3-PBA and CFMP levels in children, both below measured levels. The predicted lower levels for 3-PBA can be explained by the very low Fue value, initially proposed as conservative, but that seems to be unrealistic. The discrepancies for CFMP are mostly for the highest percentiles and require further assessments. The refined assessments included the revision of the previously proposed human biomonitoring guidance values for the general population, HBM-GV Gen Pop, following recent toxicological reevaluations, and the estimation of hazard quotients (HQs) for each individual pyrethroid and for the combined exposure to all pyrethroids. All HQs were below 1, indicating no immediate concern, but attention is required, particularly for children, with HQs in the range of 0.2–0.3 for the highly exposed group. The application of probabilistic methods offers assessments at the population level, addressing the variability in exposure and risk and providing relevant information for Public Health impact assessments and risk management prioritization.

## 1. Introduction

Pyrethroids are a large group of insecticides used worldwide for pest and vector-borne disease control in agriculture, residential areas, domestic animals, and even humans. They are synthetic analogs of pyrethrins that mimic the insecticidal activity of *Chrysanthemum cinerariaefolium* flowers and cause distress in the central nervous system by changing the dynamics of sodium cation channels in neuronal membranes [1]. It is a group of high interest and under further development, e.g., new discoveries of natural pyrethrins [2] and new formulation approaches such as nanopestices [3]. Traditionally, pyrethroids have been divided into Type I and Type II pyrethroids, but mixed modes of action have been proposed in some cases [4]. The chemistry and toxicity of pyrethroids embrace commonalities and differences [5], including significant differences in toxicity potency. Neurotoxicity is considered a common feature of pyrethroid toxicity, and the potential effects have been extended to actions at the cerebellar level, linked to functional impairment of motor coordination [6]. Developmental neurotoxicity has received particular attention [7] and is supported by epidemiological studies associating pyrethroid exposure biomarkers with neonatal effects, such as prematurity and decreased gestational age [8].

The broad variety of uses (such as pesticides, biocides, and veterinary and human pharmaceuticals) creates a complex regulatory framework for assessing the aggregate and combined exposure to pyrethroids. Considering the relevance of this pesticide group, extensive monitoring campaigns have been conducted worldwide. Human biomonitoring offers the most relevant approach for realistic human exposure quantifications, but in the case of pyrethroids, they are particularly complex as the structural similarities among the different active substances lead to common metabolites from substances with very different toxicities. Most pyrethroids are esters with a common 3-phenoxybenzyl alcohol moiety, metabolized to 3-phenoxybenzoic acid (3-PBA) or analogs, such as the fluorinated analog 4-fluor-3-phenoxybenzoic acid (4-FPBA, also abbreviated as F-3-PBA or F3PBA), frequently used as generic pyrethroid biomarkers. However, even the more specific metabolites, corresponding to the acid moiety, are frequently shared by two or more active substances, each with different toxicity potency, adding complexity to the interpretation of biomonitoring results as health indicators. In a previous work [9], a tiered retrospective risk assessment approach for assessing pyrethroid urinary metabolites was proposed. Potential concerns, particularly for children, were identified at the screening level. The probabilistic refinement suggested that the levels were close to but below the acceptability thresholds (likelihood for threshold exceedance up to 2% for the highly exposed group). Nevertheless, some refinement elements were based on assumptions supported by limited information. As pyrethroids with different toxicities have common metabolites, a key element for the interpretation of human biomonitoring data is the allocation of the metabolites to specific active substances. As a continuation of the proposed tiered risk assessment model, this study aims to develop the higher-tier retrospective risk assessment step, exploring the use of food monitoring data for developing an evidence-based approach for supporting a realistic refinement addressing variability and uncertainty. A refined assessment is proposed now, using results from prospective assessments based on food monitoring data for quantitative allocations of the contribution to the common urinary metabolite measurements for each pyrethroid.

## 2. Materials and Methods

### 2.1. Data Sources

This assessment is based on aggregated human biomonitoring data (as percentiles for each country and age group) from HBM4EU-aligned studies described in a previous publication [9]. Concentrations of pyrethroid metabolites 3-PBA, F-3-PBA, Σ(cis-DCCA + trans-DCCA) (3-(2,2-Dichlorovinyl)-2,2-dimethylcyclopropanoic acid), cis-DBCA (cis-3-(2,2-dibromovinyl)-2,2-dimethylcyclopropane-1-carboxylic acid), and CIF3CA (chlorotrifluorovinylcyclo-propane carboxylic acid (or cis-3-[2-chloro-3,3,3-trifluoroprop-1-enyl]-2,2-dimethylcyclopropanecarboxylic acid)), expressed as ug/l urine were selected. Adult and child data were processed separately.

Food monitoring data were extracted from the supporting information accompanying the EFSA annual reports [10,11,12,13,14], covering the 2016–2020 period to align the timing with the human monitoring data. Data extraction covered cyfluthrin, cypermethrin, permethrin, deltamethrin, lambda-cyhalothrin and bifenthrin. These data are expressed as percentages of the acceptable daily intake (ADI) for different diets, estimated according to the PRIMo model [15], and include three scenarios, lower, middle, and upper bounds, assuming that samples with residue levels below the limit of quantification (LOQ), have no residues, residues at half of the LOQ, or at the LOQ, respectively.

### 2.2. Data Analysis and Estimation of the Expected Contribution from Each Pyrethroid

The expected levels of urinary pyrethroid metabolites for each diet were estimated as described previously [16] using the molar urinary fractions for pyrethroids proposed by Tarazona et al. [9]. Briefly, the urinary levels expected for each metabolite at any daily dietary exposure of the parent pesticide can be estimated according to the following equation:

(1) $$\begin{array}{c} Metabolite\;urinary\;level\\ \;\;\;\;\;\;\;\;\;\;\;\;\;\;\;\;\;\;\;\;\;\;\;\;\;=\frac{parent\;pesticide\;dietary\;exposure\;\times \;Fue\;\times \;MWratio}{Body\;weight\;adjusted\;daily\;urinary\;excretion} \end{array}$$

where Fue is the molar urinary fraction of the metabolite, and MWratio is the ratio between the molecular weights of the metabolite and the parent pesticide. EFSA provides three risk estimations for each diet: the lower, middle, and upper bounds. The estimations are provided as a percentage of the ADI. This required an additional step to estimate the dietary exposure levels in mg/kg bw per day, according to the following equation:

(2) $$Dietary\;exposure=\frac{\left(EFSA\;risk\;estimation\;as\;\%ADI\times ADI\right)}{100}$$

As the EFSA estimations are provided as percentages of the ADI, if a Human BioMonitoring Guidance Value (HBM-GV) has been previously established for the same ADI, a simplified alternative estimation can be applied. The HBM-GV corresponds to the urinary levels expected for an individual exposed at the ADI level; consequently, the metabolite urinary level corresponding to the EFSA risk estimation can be estimated according to the following equation:

(3) $$Metabolite\;urinary\;level=\frac{HBM-GV}{ADI}$$

This approach was used when a suitable HBM-GV was available (see Table S1 in Supplementary Material). The HBM-GV differs between adults and children due to differences in the body weight-adjusted daily urinary excretion values. The HBM-GV for children was used for the PRIMo diets corresponding to infants, toddlers, and children; the HBM-GV for adults was used for the other PRIMo diets, including those marked as “general”. All estimations were conducted using Microsoft Excel datasheets. The graphical tools offered by this software were used for preliminary descriptive statistics analysis. The Excel file presenting the calculations of DCCA levels expected from the estimated exposure to cyfluthrin, cypermethrin, and permethrin is included as File S1 in the Supplementary Material as an example.

During the analyzed period, EFSA proposed new ADIs for cyfluthrin, cypermethrin, and permethrin; the ADI used for the PRIMo estimations were used for the urinary metabolite estimations, while the most recent ADIs were used for the risk assessment. The estimations include two metabolites for each active substance, one for the alcohol and one for the acid moiety, with the exception of bifenthrin, covered only through the acid moiety as no human biomonitoring information was available for the methylated analog of the phenoxybenzyl alcohol moiety. Table 1 summarizes the specific data used for each assessment.

For each of the 36 diets covered by PRIMo, the total predicted levels for the urinary metabolites were estimated by adding the predicted urinary levels for each common metabolite and compared with the actual human monitoring results. File S1 in the Supplementary Material presents the example for DCCA.

Then, for the common metabolites DCCA, CMFP, and 3-PBA, the percent contribution from each pyrethroid active substance was calculated by comparing the relative contribution from each pyrethroid with the total amount. Individual estimations were conducted for each diet and year. File S1 in the Supplementary Material presents the example for DCCA; then, the 10th, 50th, and 90th percentiles were estimated for the adults and children groups and for each scenario (lower, middle, and upper bounds).

For cypermethrin, permethrin, and lambda-cyhalothrin, two different metabolites were measured under HB4EU. The estimated percentages were used for calculating the molar fractions attributable to each pyrethroid from the measured urinary levels for the different population groups, using the 50th percentile of the measured human levels, the 50th percentile of the percentages attributed to each pyrethroid, and the MW and Fue values reported in Table 1.

### 2.3. Refined Risk Assessment Estimations

The estimated percentages were then applied to the human biomonitoring data (i.e., to each percentile of the aggregated HBM data for each country and age group of the HBM4EU-aligned studies mentioned in Section 2.1) for estimating the contribution of each pyrethroid, according to the following equation:

(4) $$\begin{array}{l} Urinary\;level\;of\;metabolite\;X\;originated\;from\;pyrethroid\;Y\\ \;\;\;\;\;\;\;\;\;\;\;\;\;\;\;\;\;\;\;\;\;\;\;=(Urinary\;level\;of\;metabolite\;X\\ \;\;\;\;\;\;\;\;\;\;\;\;\;\;\;\;\;\;\;\;\;\;\;\times Percent\;contribution\;of\;pyrethroid\;Y\;to\;metabolite\;X)\\ \;\;\;\;\;\;\;\;\;\;\;\;\;\;\;\;\;\;\;\;\;\;\;÷100 \end{array}$$

The calculated values were then compared with the respective HBM-GV Gen Pop for adults and children to estimate the hazard quotients (HQs) associated with the measured values for each pyrethroid and for the combined exposure to all pyrethroids contributing to the urinary metabolite levels. The HBM-GV Gen Pop proposed in the previous assessment [9] was updated according to the new ADI values proposed by EFSA if needed (see also Table S1 and File S1 in the Supplementary Material for details). The total risk for the aggregate exposure was estimated by summing up the HQs obtained for each active substance. The total pyrethroid combined risk was estimated from the sum of the HQs obtained from the most relevant metabolite of each active substance.

A Monte Carlo simulation was performed for visualizing the variability and uncertainty of the relative contributions including using Crystal Ball software with 10,000 iterations. A best-fit approach, based on the three most relevant percentiles, was applied for selecting the distribution from those provided by the Crystal Ball gallery, representing the 3-PBA population total urinary levels (based on the best fit for the 5, 50, and 95th percentiles) and the relative contribution of each pesticide (based on the best fit for the 10, 50, and 90th percentiles).

## 3. Results

### 3.1. Comparison of Food and Human Monitoring Data

EFSA PRIMo estimations are reported for different national and generic diets, in some cases distributed in age groups. Although some of the national diets could be associated with groups covered by HBM4EU aggregate data, the coverage was only partial; in addition, an exploratory assessment did not reveal consistency between the estimations using the national diets and the human monitoring distribution in the respective country. Therefore, the estimations were made by integrating all PRIMo diets as probability distributions for accounting for dietary variability at the population level, keeping exclusively the differentiation between adult and children diets.

**Figure 1:**
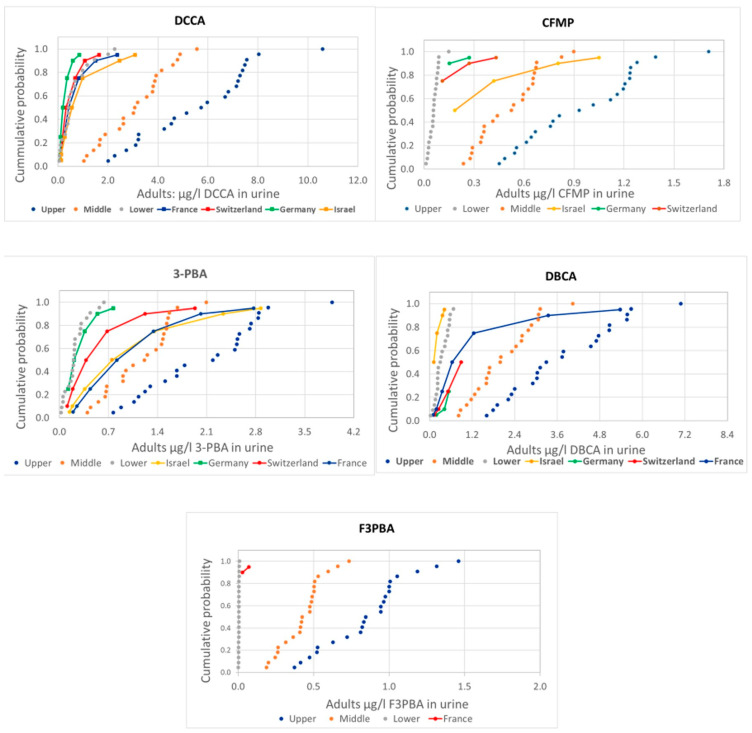
Comparison of the distribution of human urine biomonitoring data on pyrethroid metabolites from HBM4EU in adults (solid lines) with the estimations using EFSA PRIMo adult diets based on food monitoring (dotted lines).

**Figure 2:**
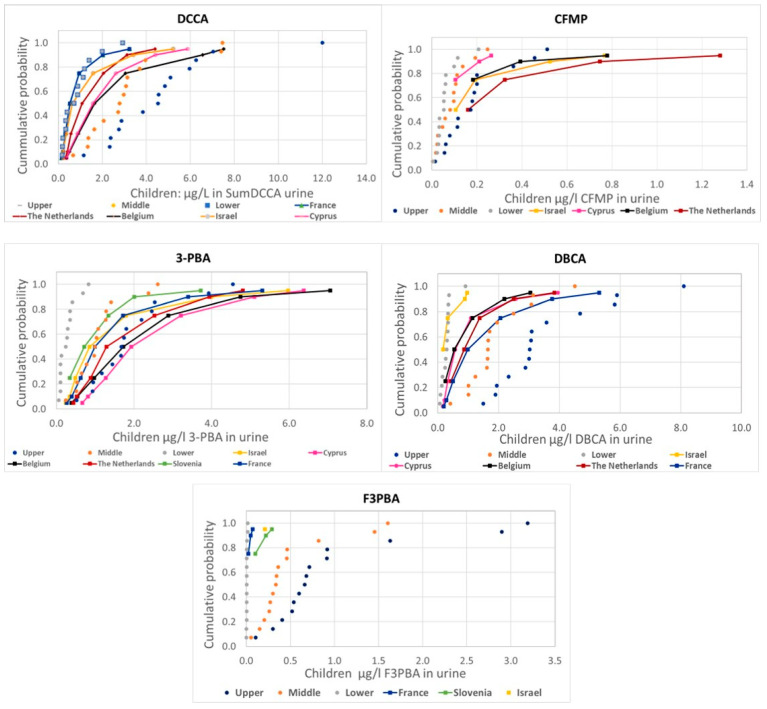
Comparison of the distribution of human urine biomonitoring data on pyrethroid metabolites from HBM4EU in children (solid lines) with the estimations using EFSA PRIMo children’s diets based on food monitoring (dotted lines).

Figure 1 and Figure 2 presents the comparison of the probability distributions of human measurements and estimations from food measurements for the different pyrethroid metabolites, covering adults and children, respectively.

There are some differences in the comparison of measured and estimated values among metabolites, but also a general consistency with actual measurements mostly around the lower range (values below LoQ assumed to be zero) or between the lower and the middle (values below LoQ assumed to be one half or the LoQ, respectively) range, except for 3-PBA and CFMP in children, with measured values above the upper estimations.

### 3.2. Estimation of Relative Contributions of Each Pyrethroid to DCCA, CMFP, and 3-BPA

Table 2 summarizes the estimated contributions as a percentage of the total estimated amount for each active substance contributing to a common metabolite. The table shows the values for the middle scenario, and the percentiles are very similar for the upper bound scenario; differences were observed for the lower bound scenario due to the consideration of all values below the LoQ as zero, but this scenario was considered of low relevance and all estimations were based on the middle scenario.

**Figure 3:**
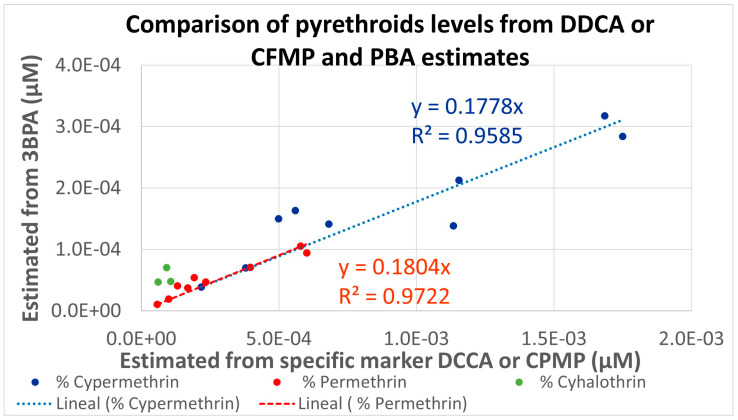
Comparison of 50th percentiles molar pyrethroids levels in the different HBM4EU populations estimated from two the selective (DDCA or CFMP) and common (3-PBA) metabolites.

As for cypermethrin, permethrin, and lambda-cyhalothrin, two different metabolites were measured under HBM4EU. The estimated percentages were used for calculating the molar fractions attributable to each pyrethroid from the measured urinary levels for the different population groups and are presented in Figure 3. Linear correlations (R^2^ higher than 0.95) are observed, but the levels estimated from 3-PBA are consistently lower than those estimated from the specific metabolites. This is related to the very conservative Fue of 0.09 used for 3-PBA; a Fue five times higher would provide similar estimations for all pyrethroids.

### 3.3. Estimations of Hazard Quotient for Each Selected Metabolite

**Figure 4:**
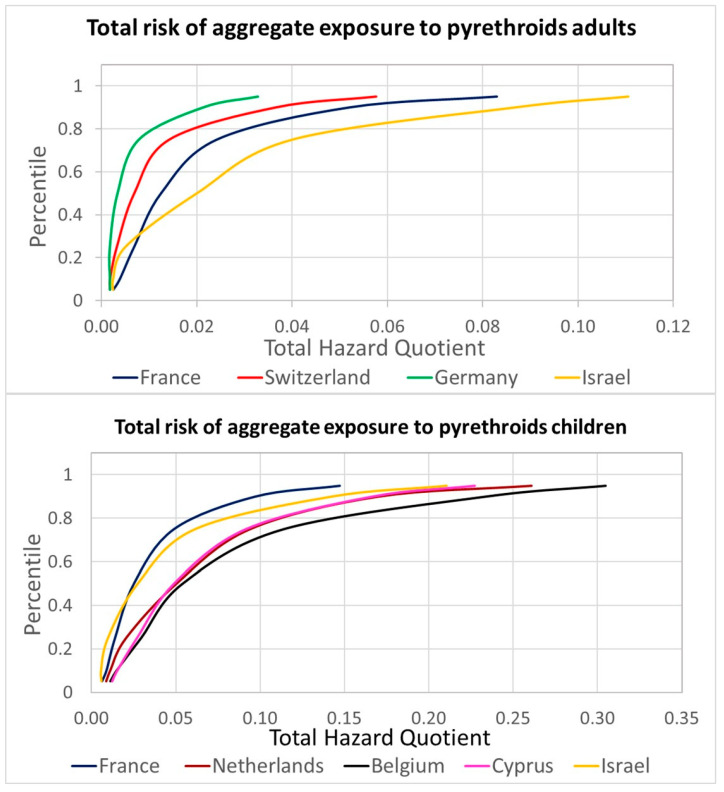
Refined total risk estimations for the aggregate exposure to pyrethroids for the aggregated HBM4EU data.

Following the new assessment by EFSA, the permethrin HBM-GV Gen Pop was updated for considering an ADI of 0.01 mg/kg body weight per day, resulting in new proposed guidance values of 64 and 97 µg DCCA/L urine for adults and children, respectively. For the refined risk assessment, the estimated contributions summarized in Table 2 were applied to the aggregated HB4EU urinary levels of pyrethroid metabolites and compared with the HBM-GV Gen Pop. All HQs for individual pyrethroids and for the cumulative assessments (sum of HQs for those contributing to the same metabolites) were below 1. Considering the 95th percentiles of the measured concentrations, cumulative HQs exceed 0.1 only for DCCA in children (maximum cumulative HQ of 0.21 for the Belgian population, and 3-PBA both in adults (maximum cumulative HQ of 0.24 for Israel) and children (maximum cumulative HQ of 0.83 for Belgium). The HQs obtained for 3-PBA were considered unrealistically high due to very conservative Fue; therefore, for the total pyrethroid combined risk, the HQs from the most selective metabolites of each active substance were selected. The results are presented in Figure 4 (see also Table S2 in the Supplementary Materials for details).

**Figure 5:**
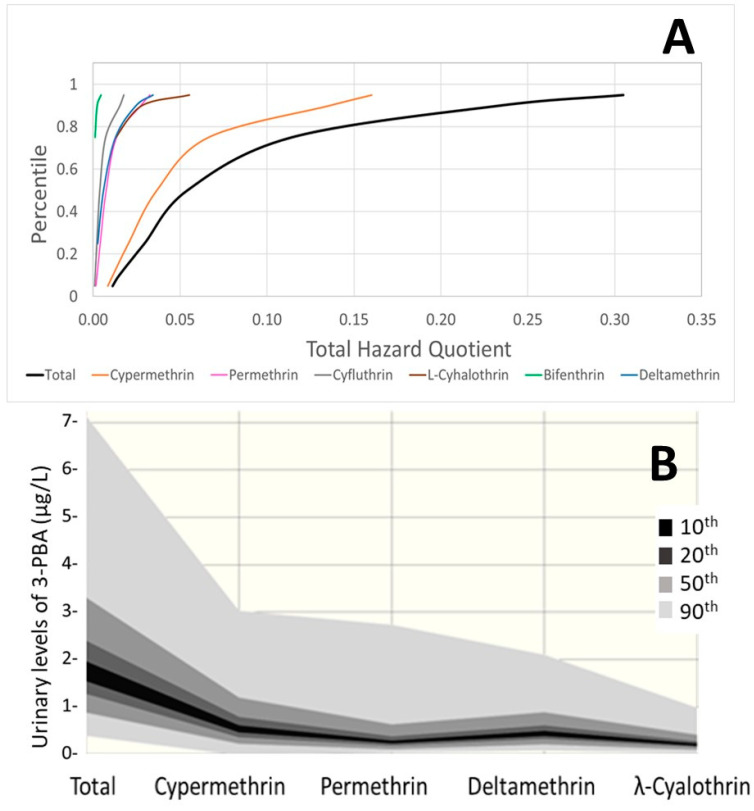
Estimated contributions of each pyrethroid for the HBM4EU Belgian children group (**A**) total aggregated and individual HQs. (**B**) Probabilistic distribution of the measured 3-PBA levels and estimated distributions for each contributing pyrethroid.

The highest risk estimation was obtained for the Belgian children group, and even the 95th percentile is well below an HQ of 1, indicating low concern. Figure 5A presents the relative contributions of each pyrethroid to the overall aggregated risk; cypermethrin is identified as the leading active substance. The combination of different years and diets provided information on the variability associated with the relevant contribution of each pyrethroid, allowing probabilistic assessments. Figure 5B presents the results of a Monte Carlo simulation for the population with a higher HQ, 3-PBA for Belgian children. The distributions used for the simulation, following a best-fitting analysis, were a Gamma distribution for the Belgian children’s urinary levels, triangular distributions for the contribution of lambda-cyhalothrin, deltamethrin, and cypermethrin, and a lognormal distribution for the contribution of permethrin. The aggregated data provided under HBM4EU, available as percentiles, were used in the simulation to build the population distribution, which was then combined with the distribution of the relative contributions as percentages estimated from EFSA PRIMo diets covering children.

## 4. Discussion

Pyrethroids are frequently included in human biomonitoring as studies on pesticides [17]. The use of human biomonitoring for realistic risk assessment offers clear advantages [18], but as previously reported, the metabolic profile of pyrethroids requires specific considerations [9]. While the common metabolites associated with the 3-phenoxybenzyl alcohol moiety facilitate screening assessments [19], the lack of unique urinary metabolites adds complexity for refining the risk, triggering our previous proposal for a tiered approach [9]. As dietary exposure is the most significant for the general population, the EU-wide information on pesticide residues in food annually reported by EFSA has been proposed by several authors as an information source for risk refinement [16,20]. The comprehensive European food consumption database [21] has not been implemented for pesticide risk assessment yet; the EFSA Model PRIMo [15] is based on national diets provided by EU member states; the overall representativeness of each diet for the European citizens has been questioned [20] and the worst-case was selected for proposing regulatory options.

Our results indicate that the distributions measured in European adult populations are, in general, within the values estimated for the low and medium scenarios for PRIMo adult diets. For children, a similar situation is observed for DCCA, DBCA, and F3-PBA, while the estimations are below measured levels for 3-PBA and CFMP. The low estimations for 3-PBA are related to the use of a low (conservative) Fue of 9%; our previous assessment [9] already suggested that this value is unrealistic, and this is further confirmed by the linear relationships, but with slopes around 0.2, shown in Figure 3. In fact, the Fue value was the most influential parameter in a sensitivity analysis of a physiologically based pharmacokinetic model (PBPK) [22]; therefore, at least as a central estimate for the population level assessments, a higher Fue value for 3-PBA should be considered. For CFMP, the comparisons indicate larger deviations for the population groups with higher measured levels; a possible explanation could be an additional contribution from sources other than the diet, reinforcing the need for aggregate exposure assessments. In fact, the role of other sources has been highlighted in a study on French adults [23], but CFMP was not included. A recent review has concluded that, in general, non-dietary exposure is much more relevant for workers than for the general population [24]; as CFMP is not a metabolite of the pyrethroids mostly used in pharmaceutical or biocidal products, further assessments are needed for understanding this result. Although co-exposure may affect the toxicokinetics of pesticides, studies have confirmed that these pyrethroid metabolites are also relevant biomarkers in cases of co-exposure at the measured levels [25,26].

Cypermethrin and deltamethrin lead the cumulative exposure in Belgian children, while the risk is mostly driven by cypermethrin due to its high toxicity. In a study on a French cohort [27], a high contribution was estimated for these two pyrethroids; in the same study, cyfluthrin also represented a significant part of the combined exposure, but this is not the case in our assessment.

The EFSA annual reports on food monitoring data have been previously used for comparing dietary estimations with real human biomonitoring data for pesticides such as chlorpyrifos with simple metabolic profiles [16]. Our results confirm their potential use for informing more complex situations. Pyrethroids are an excellent proof of concept case study as they combine complex metabolic pathways, resulting in common metabolites with significant differences in the toxicological potency of the active substances. Currently, the EFSA PRIMo is deterministic, and the offered variability is limited to the three scenarios for addressing values below the LoQ. We have addressed this limitation by conducting calculations for all of the different diets and applying inter-diet variability in our estimations. In the future, the incorporation of the comprehensive European food consumption database [28] would allow probabilistic estimations also addressing intra-diet individual variability.

Dietary exposure assessments are highly variable as a combination of both dietary differences and disparities in residue levels within the same commodity, as the use depends on agricultural needs. Under these circumstances, probabilistic risk estimations, based on a combination of full distributions or Monte Carlo simulations, offer more informative outcomes than worst-case deterministic approaches. The general good concordance in the exposure distributions obtained from food and urine monitoring further confirms the capacity of the EFSA model PRIMo for supporting regulatory risk assessments. An element for further consideration is that the actual pyrethroid-related risk for children is below but not far from the level of concern, while the levels in food, according to the EFSA risk estimations, are well below the current EU maximum residue levels (MRLs). Additional studies using national diets and food monitoring data have confirmed levels of dietary exposure below the ADI for each individual pyrethroid in Belgium [29], and similar assessments in France and Canada have been used to complement proposals for suggesting the overall benefits of increasing the consumption of fruit and vegetables [30,31]. Although pyrethroids have not been identified as the leading pesticide group in these studies [29,30,31], the expected rise in pyrethroid use due to restrictions for other insecticides could increase levels in food, reaching levels of concern for children but without exceeding the current regulatory limits. Since 2020, EFSA has published cumulative risk assessments for different effects, including neurotoxicity [32], which are clearly relevant for pyrethroids. The need to introduce cumulative assessments for setting MRLs has been highlighted by some regulatory agencies [33] and is further supported by our results.

## 5. Conclusions

The lack of unique metabolites and the diverse toxicological potency of different active substances represent major obstacles for retrospective risk assessments of pyrethroids using human monitoring levels. The comparison with prospective estimations using food monitoring data has provided good estimations for quantifying the individual contributions of each pyrethroid. The results allowed the refinement of our previous assessments, concluding that there are no immediate concerns, although levels in children should be scrutinized as an increased trend is expected, and the HQs are not far from 1.

## Figures and Tables

**Table 1:** Parameter values used for the estimations.

Active Substance	ADI	Mw	1st Metabolite	Mw	Fue	2nd Metabolite	Mw	Fue
**Deltamethrin**	0.01	505.2	DBCA	298.0	0.45	3PBA	214.2	0.09
**Cyfluthrin**	0.01	434.3	DCCA	208.1	0.36	4-F3PBA	232.2	0.47
**Cypermethrin**	0.005	416.3	DCCA	208.1	0.36	3PBA	214.2	0.09
**λ cyhalothrin**	0.0025	449.9	CFMP *	242.6	0.21	3PBA	214.2	0.09
**Bifenthrin**	0.015	422.9	CFMP *	242.6	0.21			
**Permethrin**	0.05	391.2	DCCA	208.1	0.36	3PBA	214.2	0.09

(*) CFMP is also abbreviated as CLF3CA.

**Table 2:** Relative contribution of each relevant pyrethroid to the urinary levels of DCCA, CMFP, and 3-PBA, as percentiles 10th, 50th, and 90th of the percentages for each active substance, estimated from food monitoring levels and EFSA PRIMo.

	DCCA	DCCA	DCCA	CFMP	CFMP	3-PBA	3-PBA	3-PBA	3-PBA
	% Cyfluthrin	% Cypermethrin	% Permethrin	% Cyhalothrin	% Bifenthrin	% Cyhalothrin	% Deltamethrin	% Cypermethrin	% Permethrin
Adults									
P10th	4.71	49.80	8.35	53.67	23.78	5.81	22.60	29.79	5.14
P50th	12.69	66.31	17.44	66.93	33.07	15.01	28.64	43.60	11.79
P90th	20.66	85.14	31.53	76.22	46.33	19.98	36.15	61.41	18.44
Children									
P10th	4.94	20.58	8.52	52.08	24.25	5.34	13.45	16.51	6.17
P50th	12.69	61.56	21.15	66.06	33.94	12.95	29.04	39.21	13.02
P90th	21.57	85.55	73.06	75.75	47.92	20.13	38.80	62.20	61.80

## Data Availability

The food monitoring data used for this research are published by EFSA and available at “Open EFSA” https://open.efsa.europa.eu/ accessed on 13 December 2023 and Zenodo https://zenodo.org/ both accessed on 13 December 2023. The human biomonitoring data are available at the HBM4EU Dashboard https://www.hbm4eu.eu/what-we-do/european-hbm-platform/eu-hbm-dashboard/ accessed on 13 December 2023.

## Supplementary Materials

The following supporting information can be downloaded at https://www.mdpi.com/article/10.3390/toxics12010024/s1. Table S1: HBM-GVs calculated for the selected biomarkers of each active substance; and Table S2: Summary of the refined risk characterization outcomes as Hazard Quotients (HQs) for the HBM4EU adult and children population groups; File S1. Detailed calculations were conducted for DCCA as examples of the applied methodology.
