# Supplementary material for: Applicability of Food Monitoring Data for Assessing Relative Exposure Contributions of Pyrethroids in Retrospective Human Biomonitoring Risk Estimations
Source: Toxics. 2023 Dec 28;12(1):24. doi: 10.3390/toxics12010024 (PMC10819063; doi:10.3390/toxics12010024)
Supplement: Supplementary file 1 [file toxics-12-00024-s001.zip › Pyrethroids Toxics 2023 Supplementary material Tables S1 and S2.pdf]

Applicability of food monitoring data for assessing relative exposure contributions of pyrethroids in retrospective human biomonitoring risk estimations.

Mercedes de Alba-Gonzalez[1,*], Maria Carmen González-Caballero[1] , and Jose V. Tarazona[1]

1 National Centre for Environmental Health, Instituto de Salud Carlos III, 28220 Madrid, Spain; malba@isciii.es; mcgonzalez@isciii.es; jtarazona@isciii.es

* Correspondence: Mercedes de Alba-Gonzalez malba@isciii.es

SUPPLEMENTARY MATERIAL

Table S1. HBM-GVs calculated for the selected biomarkers of each active substance

Active substance	ADI 2020	ADI before 2020	Biomarker	Fue	HBM-GV before 2020		HBM-GV 2020	
					Children	Adults	Children	Adults
Cyfluthrin	0,01	0,003	DCCA	36	19	28,5	**57**	**86**
			F3PBA	47	27,6	41,5	**84**	**126**
Cypermethrin	0,005	0,05	DCCA	36	300	450	**30**	**45**
			3PBA	9	77	116	**8**	**12**
Permethrin	0,01	0,05	DCCA	36	320	480	**64**	**96**
			3PBA	9	82	123	**16**	**25**
L-Cyhalothrin	0,025	0,025	CFMP	21	9	14	**9**	**14**
			3PBA	9	3,6	5,4	**3,6**	**5,4**
Deltamethrin	0,01	0,01	DBCA	45	89	133	**89**	**133**
			3PBA	9	13	19	**13**	**19**
Bifenthrin	0,015	0,015	CFMP	21	60	90	**60**	**90**

Table S2. Summary of the refined risk characterization outcomes, as Hazard Quotients (HQ) for the HBM4EU adult and children population groups.

		TOTAL RISK		ADULTS				
		Cypermethtrin	Permethrin	Cyfluthrin	L-Cyhalothrin	Bifenthrin	Deltamethrin	
		HQ DCCA	HQ DCCA	HQ DCCA	HQ CFMP	HQ CFMP	HQ DBCA	TOTAL HQ
	**Percentile**	Average	Average	Average				SUM
**France**	0.05	0.00141	0.0003	0.0002			0.00071	0.00
	0.10	0.00191	0.0004	0.0002			0.00142	0.00
	0.25	0.00325	0.0006	0.0004			0.00269	0.01
	0.50	0.00601	0.0011	0.0007			0.00476	0.01
	0.75	0.01148	0.0021	0.0013			0.00939	0.02
	0.90	0.02082	0.0038	0.0023			0.02518	0.05
	0.95	0.03275	0.0060	0.0037			0.04050	0.08
**Switzerland**	0.05	0.00138	0.0003	0.0002				0.00
	0.10	0.00138	0.0003	0.0002				0.00
	0.25	0.00249	0.0005	0.0003				0.00
	0.50	0.00456	0.0008	0.0005			0.00098	0.01
	0.75	0.00954	0.0018	0.0011			0.00188	0.01
	0.90	0.01490	0.0027	0.0017	0.0124	0.0011	0.00392	0.04
	0.95	0.02273	0.0042	0.0026	0.0198	0.0017	0.00671	0.06
**Germany**	0.05	0.00138	0.0003	0.0002				0.00
	0.10	0.00138	0.0003	0.0002				0.00
	0.25	0.00138	0.0003	0.0002				0.00
	0.50	0.00263	0.0005	0.0003				0.00
	0.75	0.00498	0.0009	0.0006			0.00143	0.01
	0.90	0.00831	0.0015	0.0009	0.0069	0.0006	0.00311	0.02

	0.95	0.01177	0.0022	0.0013	0.0124	0.0011	0.00407	0.03
Israel	0.05	0.00187	0.0003	0.0002				0.00
	0.10	0.00187	0.0003	0.0002				0.00
	0.25	0.00387	0.0007	0.0004				0.01
	0.50	0.00773	0.0014	0.0009	0.0084	0.0007	0.00091	0.02
	0.75	0.01367	0.0025	0.0015	0.0192	0.0017	0.00157	0.04
	0.90	0.03409	0.0063	0.0038	0.0369	0.0032	0.00273	0.09
	0.95	0.04258	0.0078	0.0048	0.0481	0.0042	0.00315	0.11

		TOTAL RISK		CHILDREN				
		Cypermethrin	Permethrin	Cyfluthrin	L-Cyhalothrin	Bifenthrin	Deltamethrin	
		HQ DCCA	HQ DCCA	HQ DCCA	HQ CFMP	HQ CFMP	HQ DBCA	TOTAL HQ
	Percentile	Average	Average	Average				SUM
France	0.05	0.0032	0.0006	0.0004			0.00223	0.01
	0.1	0.0045	0.0008	0.0005			0.00317	0.01
	0.25	0.0065	0.0012	0.0007			0.00567	0.01
	0.5	0.0109	0.0020	0.0012			0.01111	0.03
	0.75	0.0197	0.0036	0.0022			0.02332	0.05
	0.9	0.0420	0.0077	0.0047			0.04253	0.10
	0.95	0.0671	0.0124	0.0076			0.06008	0.15
Netherlands	0.05	0.0067	0.0012	0.0008				0.01
	0.1	0.0086	0.0016	0.0010				0.01
	0.25	0.0120	0.0022	0.0014			0.00494	0.02
	0.5	0.0225	0.0041	0.0025	0.0115		0.00974	0.05
	0.75	0.0426	0.0078	0.0048	0.0230		0.01554	0.09
	0.9	0.0648	0.0119	0.0073	0.0532	0.0045	0.02813	0.17
	0.95	0.0913	0.0168	0.0103	0.0913	0.0076	0.04337	0.26

**Belgium**	0.05	0.0086	0.0018	0.0009				0.01
	0.1	0.0114	0.0023	0.0012				0.01
	0.25	0.0204	0.0042	0.0021			0.00279	0.03
	0.5	0.0366	0.0076	0.0038			0.00606	0.05
	0.75	0.0663	0.0136	0.0071	0.0130	0.0011	0.01285	0.11
	0.9	0.1358	0.0274	0.0154	0.0281	0.0024	0.02469	0.23
	0.95	0.1601	0.0326	0.0176	0.0555	0.0046	0.03441	0.30
**Cyprus**	0.05	0.0080	0.0016	0.0009			0.00183	0.01
	0.1	0.0100	0.0018	0.0011			0.00244	0.02
	0.25	0.0181	0.0033	0.0020			0.00362	0.03
	0.5	0.0328	0.0060	0.0037			0.00645	0.05
	0.75	0.0543	0.0107	0.0061	0.0074	0.0006	0.01228	0.09
	0.9	0.0915	0.0191	0.0103	0.0150	0.0013	0.02907	0.17
	0.95	0.1218	0.0264	0.0138	0.0188	0.0016	0.04476	0.23
**Israel**	0.05	0.0045	0.0008	0.0005				0.01
	0.1	0.0043	0.0008	0.0005				0.01
	0.25	0.0075	0.0014	0.0008				0.01
	0.5	0.0133	0.0024	0.0015	0.0076	0.0006	0.00198	0.03
	0.75	0.0329	0.0061	0.0037	0.0138	0.0012	0.00372	0.06
	0.9	0.0707	0.0130	0.0080	0.0373	0.0031	0.01005	0.14
	0.95	0.1083	0.0199	0.0122	0.0546	0.0046	0.01095	0.21
